# On the *Lathrobium* fauna of the Nanling National Nature Reserve, southern China (Coleoptera, Staphylinidae, Paederinae)

**DOI:** 10.3897/zookeys.1054.68991

**Published:** 2021-08-03

**Authors:** Xiao-Bin Lin, Yi-Dan Zhen, Zhong Peng

**Affiliations:** 1 Department of Biology, Shanghai Normal University, 100 Guilin Road, Shanghai, 200234, China Shanghai Normal University Shanghai China

**Keywords:** Guangdong, new species, taxonomy

## Abstract

Material of the paederine genus *Lathrobium* Gravenhorst, 1802 from the Nanling National Nature Reserve, southern China, is examined. Four species are identified, one of them described previously. Three species are described and illustrated for the first time, all of them micropterous and locally endemic: *L.yangyihani* Lin & Peng **sp. nov.**, *L.jiaxingyangi* Lin & Peng **sp. nov.**, and *L.wangxingmini* Lin & Peng **sp. nov.** The female sexual characters of *L.guangdongense* Peng & Li, 2014 are described and illustrated for the first time. Including the new taxa, 224 *Lathrobium* species are currently known from mainland China.

## Introduction

From mainland China, 221 species of the genus *Lathrobium* Gravenhorst have been reported, with the vast majority of them locally endemic (Zhao and Peng 2021). One micropterous species was previously recorded from Guangdong and six species from Hunan: *L.guangdongense* Peng & Li, 2014 (Guangdong: Nanling), *L.badagongense* Peng & Li, 2014 (Hunan: Badagong Shan), *L.bamianense* Peng & Li, 2016 (Hunan: Bamian Shan), *L.fumingi* Peng & Li, 2016 (Hunan: Bamian Shan), *L.hunanense* Watanabe, 2011 (Hunan: Longshan), *L.jinyuae* Peng & Li, 2016 (Hunan: Nanfengmian; Jiangxi: Bijia Shan), and *L.kishimotoi* Watanabe, 2011 (Hunan: Longshan) (Watanabe 2011; Peng et al. 2014, 2016).

Covering an area of 58,400 hm^2^, the Nanling National Nature Reserve is situated at the border between Guangdong and Hunan provinces and includes the easternmost part of the Nanling range. The highest peak of the Nanling National Nature Reserve is the Shikengkong at 1,902 m (Liu et al. 2018).

In recent years, we conducted several collecting trips and obtained numerous *Lathrobium* specimens. Four species were recognized, including three new species and the previously unknown females of *L.guangdongense*.

## Material and methods

The following abbreviations are used in the text, with all measurements in millimeters:

Body length (**BL**) from the anterior margin of the mandibles (in resting position) to the abdominal apex; length of forebody (**FL**) from the anterior margin of the mandibles to the posterior margin of the elytra; head length (**HL**) from the anterior margin of the frons to the posterior margin of the head; head width (**HW**): maximum width of head; length of antenna (**AnL**); length of pronotum (**PL**) along midline; maximum width of pronotum (**PW**); elytral length (**EL**) at the suture from the apex of the scutellum to the posterior margin of the elytra (at the sutural angles); length of aedeagus (**AL**) from the apex of the ventral process to the base of the aedeagal capsule.

The type material is deposited in the Insect Collection of Shanghai Normal University, Shanghai, China (**SNUC**).

## Results

### Lathrobium yangyihani

Taxon classificationAnimaliaColeopteraStaphylinidae

Lin & Peng sp. nov.

177BA24D-4318-5222-A8F5-E1279D94DEAA

http://zoobank.org/8FACCDF3-F4DA-4688-99D9-FA3819B4DD2E

Figures 1A , 2 , 5

#### Type material.

***Holotype*:** ♂, labeled ‘China: Guangdong Prov., Ruyuan County, Nanling National Nature Reserve, Hamashi, 24°55'37"N, 112°59'21"E, 1,750 m, 01.V.2015, Peng, Tu & Zhou leg.’ (SNUC). ***Paratypes***: 3 ♀♀, same data as the holotype (SNUC); 2 ♀♀, labeled ‘China: Guangdong Prov., Ruyuan County, Guangdong Di Yi Feng, 24°55'29.62"N, 112°59'31.42"E, 1,538–1,784 m, 28.VI.2020, Xia, Zhang, Yin & Lin leg.’ (SNUC); 2 ♀♀, labeled ‘China: Guangdong Prov., Ruyuan County, Nanling National Nature Reserve, Shikengkong, 24°55'33"N, 112°59'29"E, 1,820 m, 30.IV.2015, Peng, Tu & Zhou leg.’ (SNUC); 1 ♀, labeled ‘China: Guangdong Prov., Ruyuan County, Nanling National Nature Reserve, Shikengkong, 24°55'38"N, 112°59'30"E, 1,850 m, 27.IV.2015, Peng, Tu & Zhou leg.’ (SNUC); 1 ♀, labeled ‘China: Guangdong Prov., Ruyuan County, Nanling National Nature Reserve, Laopengyidui, 24°56'21"N, 113°01'21"E, 1,260 m, 02.V.2015, Peng, Tu & Zhou leg.’ (SNUC); 1 ♂, 1 ♀, labeled ‘China: Guangdong Prov., Shixing County, Chebaling, 24°43'12.27"N, 114°11'18.03"E, 904–1,004 m, 23.VI.2020, Xia, Zhang, Yin & Lin leg.’ (SNUC).

#### Description.

Measurements (in mm) and ratios: BL 5.34–6.67, FL 2.19–2.67, HL 0.76–0.98, HW 0.78–0.87, AnL 1.49–1.74, PL 1.00–1.09, PW 0.82–0.93, EL 0.41–0.65, AL 0.81–0.83, HL/HW 0.97–1.13, HW/PW 0.90–1.00, HL/PL 0.75–0.92, PL/PW 1.15–1.27, EL/PL 0.41–0.60.

**Figure 1.**
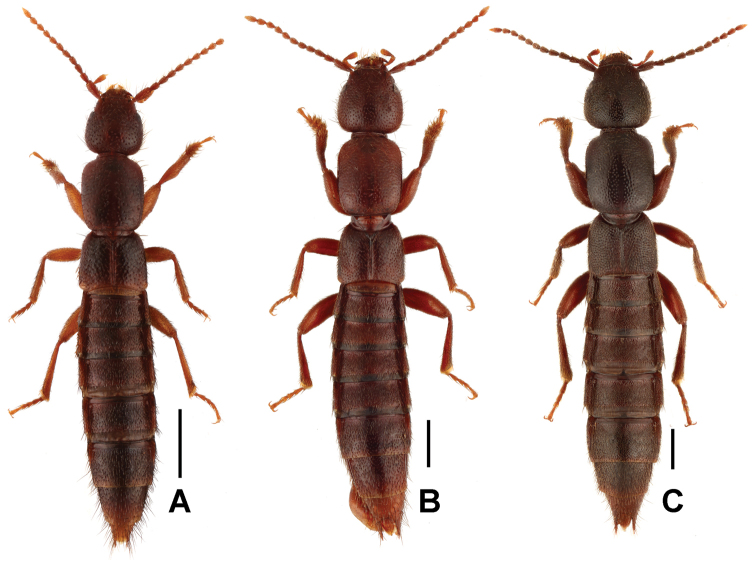
Male habitus of *Lathrobium* spp. **A***L.yangyihani***B***L.jiaxingyangi***C***L.wangxingmini*. Scale bars: 1.0 mm.

Habitus as in Figure 1A. Body brown, legs, and antennae light brown.

Head approximately as long as broad; punctation moderately coarse and sparse, sparser in median dorsal portion; interstices with shallow microreticulation; eyes small and composed of approximately 40 ommatidia.

Pronotum nearly parallel-sided; punctation somewhat sparser than that of head; impunctate midline broad; interstices without microsculpture.

Elytral punctation moderately dense, shallow, and ill-defined. Hind wings completely reduced.

Abdomen with fine and moderately sparse punctation, that of tergite VII somewhat sparser than that of anterior tergites; interstices with shallow microsculpture; posterior margin of tergite VII without palisade fringe.

**Figure 2.**
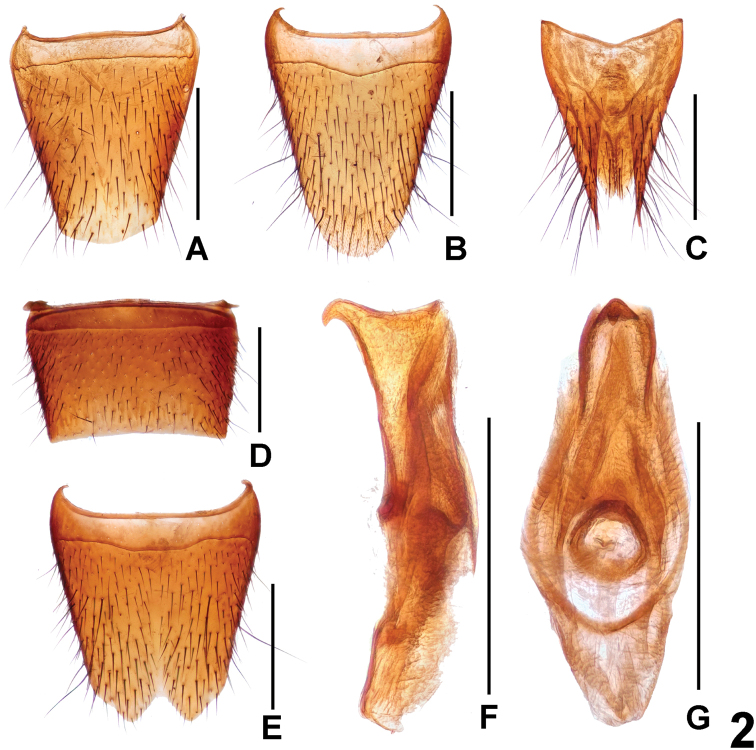
*Lathrobiumyangyihani*. **A** female tergite VIII **B** female sternite VIII **C** female tergites IX–X. **D** male sternite VII **E** male sternite VIII **F** aedeagus in ventral view **G** aedeagus in lateral view. Scale bars: 0.5 mm.

**Male.** Sternites III–VI unmodified; sternite VII (Fig. 2D) with very shallow postero-median impression without distinctly modified setae, posterior margin weakly concave in the middle; sternite VIII (Fig. 2E) with symmetric, subtriangular emargination and shallow impression; aedeagus as in Figure 2F and G, with stout ventral process of highly distinctive shape; dorsal plate with moderately long apical portion and very short basal portion; internal sac without sclerotized spines.

**Female.** Posterior margin of tergite VIII (Fig. 2A) strongly convex. Posterior margin of sternite VIII (Fig. 2B) strongly convex and with moderately dense micropubescence; tergite IX (Fig. 2C) with very short, medially undivided antero-median portion and moderately long postero-lateral processes; tergite X (Fig. 2C) 2.5 times as long as antero-median portion of tergite IX.

#### Comparative notes.

**Figure 3.**
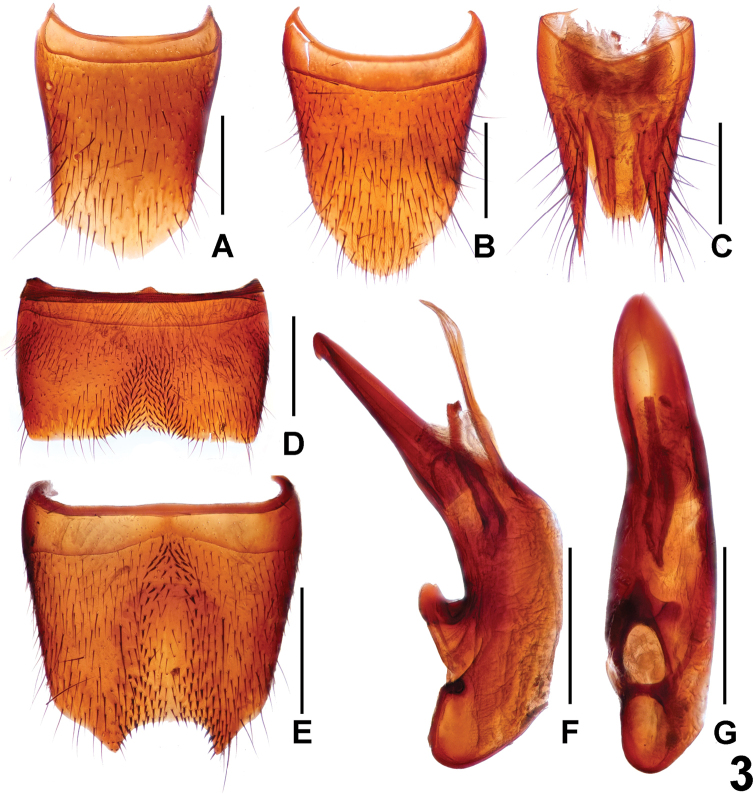
*Lathrobiumguangdongense* (**A–C**) and *L.jiaxingyangi* (**D–G**) **A** female tergite VIII **B** female sternite VIII **C** female tergites IX–X. **D** male sternite VII **E** male sternite VIII **F** aedeagus in ventral view **G** aedeagus in lateral view. Scale bars: 0.5 mm.

The new species resembles *L.guangdongense* Peng & Li, 2014 in habitus and the similarly derived morphology of Sternites VII–VIII but differs from this species by the stouter ventral process and the simple internal sac of the aedeagus. For illustrations of *L.guangdongense*, see Figure 3A–C and Peng et al. (2014).

#### Etymology.

The species is named after Yi-Han Yang, who supported us on our field trips.

#### Distribution and natural history.

The species was found in six adjacent localities in western Ruyuan County to southeastern Shixing County. A specimen was sifted, together with *L.guangdongense*, from moist leaf litter of a secondary mixed and deciduous forest at an altitude of 1,260 m (Fig. 5).

### Lathrobium guangdongense

Taxon classificationAnimaliaColeopteraStaphylinidae

Peng & Li, 2014

FFD27766-BC12-5A82-B1EC-7A30EE0E0610

Figure 3A–C

#### Material studied.

3 ♂♂, 6 ♀♀, China: Guangdong Prov., Ruyuan County, Nanling National Nature Reserve, Laopengyidui, 24°56'21"N, 113°01'21"E, 1,260 m, 02.V.2015, Peng, Tu & Zhou leg. (SNUC); 4 ♂♂, 3 ♀♀, China: Guangdong Prov., Ruyuan County, Nanling National Nature Reserve, Laopengkeng, 24°56'29"N, 113°00'27"E, 1,360 m, 29.V.2015, Peng, Tu & Zhou leg. (SNUC); 1 ♀, China: Guangdong Prov., Ruyuan County, Nanling National Nature Reserve, Walkway, 24°55'57"N, 113°00'18"E, 1,220 m, 28.IV.2015, Peng, Tu & Zhou leg. (SNUC); 2 ♂♂, China: Guangdong Prov., Ruyuan County, Nanling National Nature Reserve, Disilindao, 24°55'47"N, 112°59'50"E, 1,500 m, 05.V.2015, Peng, Tu & Zhou leg. (SNUC); 1 ♂, China: Hunan Prov., Yizhang County, Mangshan Nature reserve, 24°56'26"N, 112°59'18"E, 1400 m, 26.IV.2015, Peng, Tu & Zhou leg. (SNUC); 3 ♂♂, 4 ♀♀, China: Guangdong Prov., Ruyuan County, Nanling National Nature reserve, 24°55'43.67"N, 113°00'58.50"E, 1,020 m, sifted, 27.VI.2020, Lin, Xia, Yin & Zhang leg. (SNUC); 1 ♂, 2 ♀♀, China: Hunan Prov., Xinning County, Shunhuang Shan Nature Reserve, 26°22'33.82"N, 110°59'32.11"E, 1,112 m, sifted, 24.VIII.2020, leaf litter, sifted, Chong Li leg. (SNUC); 2 ♂♂, 1 ♀, China: Guangdong Prov., Ruyuan County, Nanling Nature reserve, 24°55'42.9"N, 113°00'59.05"E, 1,020–1,250 m, 04.V.2021, Hu, Lin, Zhou & Li leg. (SNUC); 2 ♀♀, China: Guangdong Prov., Ruyuan County, Nanling Nature reserve, 24°56'16.20"N, 113°00'8.43"E, 980–1,350 m, 01.V.2021, Hu, Lin, Zhou & Li leg. (SNUC); 2 ♂♂, 2 ♀♀, China: Guangdong Prov., Ruyuan County, Nanling Nature reserve, Xiaohuangshan, 24°53'44.7"N, 113°01'26.9"E, 1,270–1,570 m, 02.V.2021, Hu, Lin, Zhou & Li leg. (SNUC).

#### Comment.

The original description is based on four males. The previously unknown female sexual characters are as follows: posterior margin of tergite VIII (Fig. 2A) convex; posterior margin of sternite VIII (Fig. 2B) strongly convex and with moderately dense micropubescence; tergite IX (Fig. 2C) with median suture and long postero-lateral processes; tergite X (Fig. 2C) nearly reaching anterior margin of tergite IX. The above records from Hunan represent new province records. For illustrations of the habitus and the male sexual characters, see Peng et al. (2014).

### Lathrobium jiaxingyangi

Taxon classificationAnimaliaColeopteraStaphylinidae

Lin & Peng sp. nov.

B171589A-3AFE-56FD-AB35-54BF48C13F1A

http://zoobank.org/A2AC29DE-EA0E-438B-BEE4-1D131CFE0B1F

Figures 1B , 3D–G , 6

#### Type material.

***Holotype*:** ♂, labeled ‘China: Guangdong Prov., Ruyuan County, Nanling National Nature Reserve, Shikengkong, 24°55'33"N, 112°59'29"E, 1,820 m, 30.IV.2015, Peng, Tu & Zhou leg.’ (SNUC). ***Paratypes***: 1 ♂, same data, but ‘24°55'38"N, 112°59'30"E, 1,850 m, 27.IV.2015, Peng, Tu & Zhou leg’ (SNUC).

#### Description.

Measurements (in mm) and ratios: BL 7.95–8.34, FL 3.12–3.24, HL 1.09–1.14, HW 1.09, AnL 1.99–2.16, PL 1.34–1.37, PW 1.09–1.14, EL 0.68–0.73, AL 1.55, HL/HW 1.00–1.04, HW/PW 0.96–1.00, HL/PL 0.82–0.83, PL/PW 1.20–1.22, EL/PL 0.51–0.53.

Habitus as in Figure 1B. Body dark reddish brown, legs reddish brown, antennae dark to light reddish brown.

Head punctation moderately fine and moderately dense, not sparser in median dorsal portion; interstices with shallow microsculpture. Eyes moderately small and composed of approximately 60 ommatidia.

Pronotum nearly parallel-sided; punctation somewhat sparser than that of head; impunctate midline broad; interstices without microsculpture.

Elytral punctation moderately dense and shallow. Hind wings completely reduced.

Abdomen with fine and moderately dense punctation, that of tergite VII somewhat sparser than that of anterior tergites; interstices with shallow, but distinct microsculpture; posterior margin of tergite VII without palisade fringe.

**Male.** Sternites III–VI unmodified; sternite VII (Fig. 3D) strongly transverse and symmetric, with shallow median impression with modified short black setae, posterior margin broadly and very weakly concave; sternite VIII (Fig. 3E) approximately as long as broad, with moderately extensive median impression posteriorly, this impression with numerous distinctly modified, stout black setae, posterior excision relatively deep; aedeagus as in Figure 3F and G; ventral process weakly hooked apically in lateral view; dorsal plate with long apical portion and very short basal portion; internal sac with one sclerotized spine.

**Female.** Unknown.

#### Comparative notes.

*Lathrobiumjiaxingyangi* resembles *L.wangxingmini* sp. nov. in having the similarly derived chaetotaxy of the asymmetric male sternite VIII and the long dorsal plate of the aedeagus. It is distinguished from *L.wangxingmini* by the lighter coloration, the smaller body size, the chaetotaxy of the male sternite VII, the shallower posterior excision of the male sternite VIII, and the morphology of the aedeagus (shape of ventral process; internal sac with one shorter sclerotized spine).

#### Etymology.

The species is named after Xing-Yang Jia, who supported us on our field trips.

#### Distribution and natural history.

The type locality is situated in the Nanling National Nature Reserve to western Ruyuan County, eastern Guangdong. The specimens were sifted from leaf litter and grass roots in shrub habitats at an altitude of 1,850 m (Fig. 6).

### Lathrobium wangxingmini

Taxon classificationAnimaliaColeopteraStaphylinidae

Peng & Lin sp. nov.

2478B758-4B35-5CE5-8C26-B029AF1F493E

http://zoobank.org/A47F4DF3-D990-4DEB-A7C9-0F2F3891E52D

Figures 1C , 4 , 7

#### Type material.

***Holotype*:** ♂, labeled ‘China: Guangdong Prov., Ruyuan County, Nanling National Nature Reserve, Walkway, 24°55'57"N, 113°00'18"E, 1,220 m, 28.IV.2015, Peng, Tu & Zhou leg.’ (SNUC). ***Paratypes***: 1 ♀, labeled ‘China: Guangdong Prov., Ruyuan County, Nanling National Nature Reserve, Shikengkong, 24°55'33"N, 112°59'29"E, 1,820 m, 30.IV.2015, Peng, Tu & Zhou leg.’ (SNUC); 1 ♀, labeled ‘China: Guangdong Prov., Ruyuan County, Nanling National Nature Reserve, Shikengkong, 24°55'38"N, 112°59'30"E, 1,850 m, 27.IV.2015, Peng, Tu & Zhou leg’ (SNUC); 1 ♀, labeled ‘China: Hunan Prov., Yizhang County, Mangshan Nature reserve, 24°56'26"N, 112°59'18"E, 1,400 m, 26.IV.2015, Peng, Tu & Zhou leg.’ (SNUC); 1 ♂, 2 ♀♀, labeled ‘China: Guangdong Prov., Ruyuan County, Nanling National Nature Reserve, 1,090 m, 18.VI.2007, Huang & Xu leg.’ (SNUC).

#### Description.

Measurements (in mm) and ratios: BL 8.62–10.56, FL 3.48–4.27, HL 1.30–1.67, HW 1.30–1.52, AnL 2.32–2.70, PL 1.57–1.85, PW 1.33–1.57, EL 0.61–0.83, AL 2.25, HL/HW 1.00–1.15, HW/PW 0.96–0.98, HL/PL 0.82–0.95, PL/PW 1.18–1.19, EL/PL 0.39–0.45.

**Figure 4.**
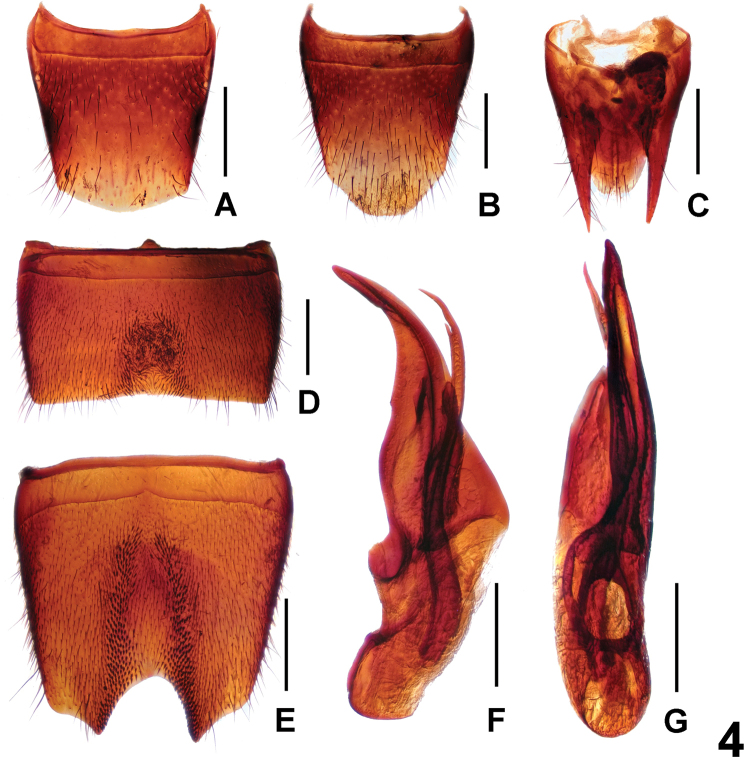
*Lathrobiumwangxingmini*. **A** female tergite VIII **B** female sternite VIII **C** female tergites IX–X. **D** male sternite VII **E** male sternite VIII **F** aedeagus in ventral view **G** aedeagus in lateral view. Scale bars: 0.5 mm.

Habitus as in Fig. 4C. Body blackish brown, legs brown, antennae dark to light brown.

Head transverse; punctation coarse and dense, sparser in median dorsal portion; interstices with distinct microsculpture.

Pronotum nearly parallel-sided; punctation somewhat sparser than that of head; impunctate midline broad; interstices without microsculpture. Eyes moderately big and composed of approximately 80 ommatidia.

Elytral punctation dense and defined. Hind wings completely reduced. Protarsi without appreciable sexual dimorphism, distinctly dilated.

Abdomen with fine and dense punctation, punctation of tergite VII slightly less dense than that of anterior tergites; interstices with shallow microsculpture; posterior margin of tergite VII without palisade fringe.

**Male.** Sternites III–VI unmodified; sternite VII (Fig. 4D) strongly transverse, with shallow median impression posteriorly with numerous distinctly modified, short black setae, posterior margin nearly truncate; sternite VIII (Fig. 4E) strongly modified and of distinctive shape and chaetotaxy, with deep and extensive median impression, middle of this impression with unmodified pubescence, laterally with dense short and very stout black setae, posterior margin deep and weakly asymmetric; aedeagus as in Figure 4F and G; ventral process asymmetric and apically acute; dorsal plate with long apical portion and very short basal portion; internal sac with one long sclerotized spine.

**Female.** Posterior margin of tergite VIII (Fig. 4A) strongly convex. Posterior margin of sternite VIII (Fig. 4B) strongly convex and with moderately dense micropubescence; tergite IX (Fig. 4C) with short antero-median portion and slender postero-lateral processes; tergite X (Fig. 4C) 2.5 times as long as antero-median portion of tergite IX.

#### Comparative notes.

*Lathrobiumwangxingmini* resembles *L.jiaxingyangi* sp. nov. in having the similarly derived chaetotaxy of the asymmetric male sternite VIII and the long dorsal plate of the aedeagus. It is distinguished from *L.jiaxingyangi* by the darker coloration, the larger body size, more dense short black setae in the impression of the male sternite VII, the deeper posterior excision of the male sternite VIII and the morphology of the aedeagus (shape of ventral process; internal sac with one longer sclerotized spine).

#### Etymology.

The species is dedicated to Xing-Min Wang, who supported us on our field trips.

#### Distribution and natural history.

The species was found in five adjacent localities in western Ruyuan County to southern Yizhang County. Some specimens were sifted from the leaf litter of mixed deciduous forests at an altitude of 1,090 m (Fig. 7).

**Figures 5–7.**
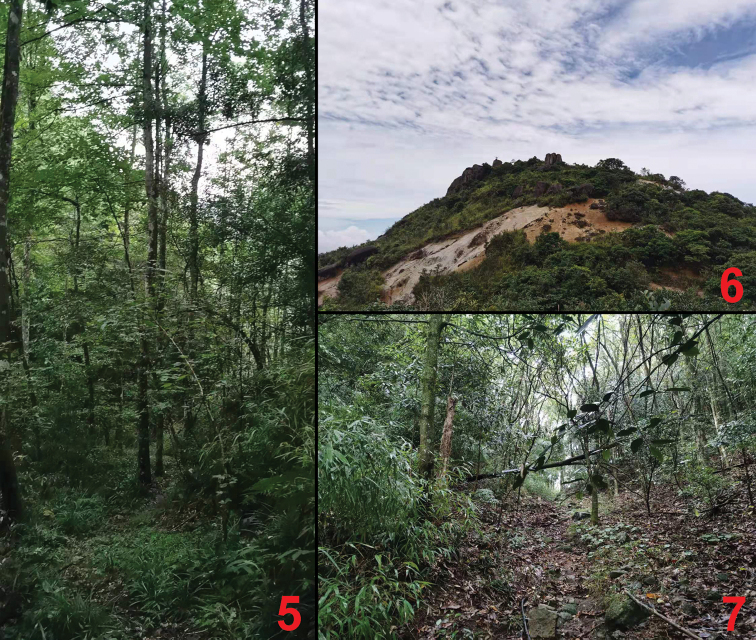
Collecting sites on the Nanling National Nature Reserve **5** type localities of *Lathrobiumyangyihani* (1,260 m) **6** type locality of *Lathrobiumjiaxingyangi* (1,850 m) **7** type locality of *Lathrobiumwangxingmini* (1,090 m).

## Supplementary Material

XML Treatment for Lathrobium yangyihani

XML Treatment for Lathrobium guangdongense

XML Treatment for Lathrobium jiaxingyangi

XML Treatment for Lathrobium wangxingmini
